# *IGF2*/*H19* hypomethylation is tissue, cell, and CpG site dependent and not correlated with body asymmetry in adolescents with Silver-Russell syndrome

**DOI:** 10.1186/1868-7083-4-15

**Published:** 2012-09-18

**Authors:** Kai Kannenberg, Karin Weber, Cathrin Binder, Christina Urban, Hans-Joachim Kirschner, Gerhard Binder

**Affiliations:** 1 University Children’s Hospital Tübingen Pediatric Endocrinology, 72076, Tübingen, Germany; 2 Department of Pediatric Surgery, University Children’s Hospital Tübingen, 72076, Tübingen, Germany

**Keywords:** Silver-Russell syndrome, DNA methylation, Body asymmetry, Skin fibroblast culture, IGF-II

## Abstract

**Background:**

Silver-Russell syndrome (SRS) is characterized by severe intrauterine and postnatal growth failure and frequent body asymmetry. Half of the patients with SRS carry a DNA hypomethylation of the imprinting center region 1 (ICR1) of the insulin-like growth factor 2 (*IGF2*)/*H19* locus, and the clinical phenotype is most severe in these patients. We aimed to elucidate the epigenetic basis of asymmetry in SRS and the cellular consequences of the ICR1 hypomethylation.

**Results:**

The ICR1 methylation status was analyzed in blood and in addition in buccal smear probes and cultured fibroblasts obtained from punch biopsies taken from the two body halves of 5 SRS patients and 3 controls. We found that the ICR1 hypomethylation in SRS patients was stronger in blood leukocytes and oral mucosa cells than in fibroblasts. ICR1 CpG sites were affected differently. The severity of hypomethylation was not correlated to body asymmetry. *IGF2* expression and IGF-II secretion of fibroblasts were not correlated to the degree of ICR1 hypomethylation. SRS fibroblasts responded well to stimulation by recombinant human IGF-I or IGF-II, with proliferation rates comparable with controls. Clonal expansion of primary fibroblasts confirmed the complexity of the cellular mosaicism.

**Conclusions:**

We conclude that the ICR1 hypomethylation SRS is tissue, cell, and CpG site specific. The correlation of the ICR1 hypomethylation to *IGF2* and *H19* expression is not strict, may depend on the investigated tissue, and may become evident only in case of more severe methylation defects. The body asymmetry in juvenile SRS patients is not related to a corresponding ICR1 hypomethylation gradient, rendering more likely an intrauterine origin of asymmetry. Overall, it may be instrumental to consider not only the ICR1 methylation status as decisive for *IGF2*/*H19* expression regulation.

## Background

Silver-Russell syndrome (SRS; OMIM 180860) is a sporadically occurring, genetically and clinically heterogeneous disorder. It is diagnosed on the basis of the combination of intrauterine growth retardation, severe short stature, characteristic triangular face, relative macrocephaly, body asymmetry, underweight, and several minor abnormalities [1-3]. The relative limb length differences in asymmetric SRS patients are present at birth and stay stable during the growth process [4]. Short stature in SRS can be treated with pharmacological doses of recombinant growth hormone [5]. There is no apparent hormone deficiency. In contrast, insulin-like growth factor (IGF)-I and IGF-II, and insulin-like growth factor binding protein 3 (IGFBP-3) serum levels are frequently inappropriately high in relation to the short stature, suggesting some kind of IGF-I insensitivity [6,7].

In SRS, hypomethylation of the paternal allele of the telomeric imprinting center region 1 (ICR1, also named H19DMR) within the *IGF2*/*H19* locus on 11p15 can be found in approximately 50% of the patients [8]. The clinical phenotype is most severe in these SRS patients [7,9-11]. Since in general, no complete ICR1 demethylation is observed, a mosaic distribution of the defect is assumed. The centromeric imprinting center region 2 (ICR2) within the *KCNQ1* gene on 11p15 is hypomethylated in less than 5% of ICR1 hypomethylated SRS patients [12,13]. Maternal uniparental disomy of chromosome 7 is present in roughly 10% of SRS cases [14]; the remaining cases are of unknown etiology.

ICR1 controls the imprinting of *IGF2*[15,16] on the paternal allele and of *H19* on the maternal allele. The *IGF2*-encoded IGF-II is an important growth factor for intrauterine development [17,18]. *H19* encodes a non-translated RNA whose function is unknown [19-21]. The ICR1 hypomethylation of the paternal allele found in SRS is thought to result in a relevant suppression of the normal *IGF2* expression. The hypomethylated paternal ICR1 enables binding of the insulator protein CTCF (CCCTC-binding factor) which suppresses *IGF2* and promotes *H19* expression via relocation of enhancer elements distal to *H19* from the *IGF2* promotor to the *H19* promotor [22-24]. An imprinting defect in the opposite direction, resulting in *IGF2* overexpression in response to ICR1 hypermethylation, is present in a subgroup of patients with the overgrowth syndrome described by Beckwith and Wiedemann (BWS; OMIM 130650) [25-27].

In contrast to the genetic knowledge on the etiology of SRS, experimental data on cellular downstream effects of the epimutations are scarce. Reduced *IGF2* expression was reported in skin fibroblasts from two relevant SRS patients [8] and in placentas from three newborn babies with SRS and ICR1 hypomethylation [28]. However, serum IGF-II levels of children with SRS are normal [6,9] which may reflect the non-imprinted, biallelic postnatal *IGF2* expression in the liver [29].

This study was undertaken to test the following three hypotheses. A) The body asymmetry in adolescent SRS patients with ICR1 hypomethylation is reflected by an ICR1 methylation gradient between the short and the long body side, with stronger hypomethylation at the shorter side. B) On the cellular level, ICR1 hypomethylation leads to reduced *IGF2* and enhanced *H19* expression. C) IGF-I insensitivity contributes to the etiology of SRS in ICR1 hypomethylated patients by reducing cell proliferation in response to IGF-I stimulation. Our experimental approach was to examine the degree of ICR1 hypomethylation in the tissue of the two asymmetric halves of children with SRS and to establish a fibroblast culture model system from bilaterally taken skin biopsies of SRS patients for the analysis of the relationship between ICR1 methylation status, *IGF2/H19* expression, and cell proliferation.

## Results

### ICR1 hypomethylation in various tissues and in both body halves

**Figure 1:**
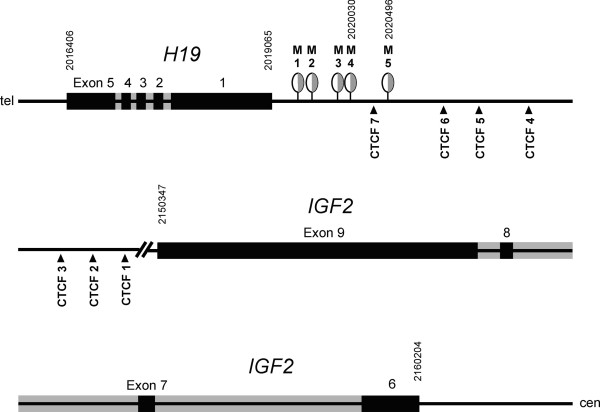
**Genomic location of MS-MLPA ICR1 sites M1 to M5 and of CTCF binding sites 1 to 7.** Genomic position (upright numbers) of CpG sites M1-5 are shown according to basic local alignment search tool (BLAST) search with the relevant sequences provided by the manufacturer (see also Additional file 1). Exon sequences were obtained from NM_000612.4 (*IGF2*) and NR_002196.1 (*H19*) at NCBI Nucleotide and matched against human genome build GRCh37 from the Genome Reference Consortium accessible at http://www.ensembl.org. CCCTC-binding factor (CTCF) site sequences from Bell *et al*. [22] were matched to the genomic sequence by BLAST search (Additional file 1). Figure adapted and modified from Kannenberg *et al*. [31]. MS-MLPA, methylation-specific multiple ligation-dependent probe amplification.

For the comparison of the degree and the characteristics of ICR1 hypomethylation in different tissues we examined blood leukocytes, cells from the oral mucosa (buccal smears) and cultured fibroblasts (forearm skin biopsies). Smears and biopsies were taken from both sides of the body in each individual (Table 1) to search for epigenetic or functional differences in the two asymmetrically developed halves of the body in SRS. In this first comparison, results are the mean of the methylation values of the CpG sites M1-M5 (for genomic location see Figure 1 and Additional file 1) and are used as the parameter to characterize the degree of methylation of ICR1. DNA methylation analysis was done by methylation-specific multiplex ligation-dependent probe amplification (MS-MLPA).

**Table 1:** Clinical characteristics of the patients with Silver-Russell syndrome (SRS)

**Patient**	**Unit**	**S1**	**S2**	**S3**	**S4**	**S5**
Duration of gestation	weeks	40	39	39	34	39
Birth length	cm (SDS^a^)	40 (−6.01)	38 (−6.14)	45 (−2.35)	34 (−4.52)	44 (−3.18)
Birth weight	g (SDS^a^)	1560 (−5.68)	1380 (−5.35)	1960 (−3.46)	955 (−3.81)	1800 (−4.23)
Triangular face		+	+	+	+	+
Body asymmetry		+	+	+	+	+
Side of shorter limb		right	left	left	right	left
Sex^b^		♂	♀	♀	♀	♂
Age	y	15.0	16.6	9.2	9.7	16.3
Blood probe		+	+	+	+	+
Buccal smear probes		+	+	+	+	+
Skin biopsies		+	+	+	-	+

^a^Standard deviation scores according to Niklasson *et al*. [30].

^b^♂:male, ♀:female.

**Figure 2:**
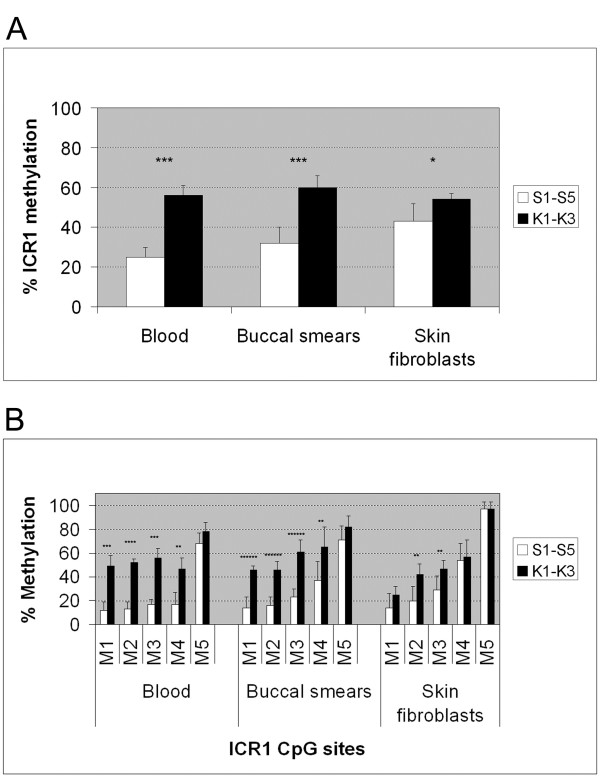
**Imprinting center region 1** (**ICR1) hypomethylation in patients with Silver-Russell syndrome (SRS) in different tissues.** The mean ICR1 (**A**) and single ICR1 sites M1 to M5 (**B**) methylation values of SRS patients S1 to S5 and control patients K1 to K3 are plotted for the different tissues investigated. *P < 0.05, ** to ******P < 0.01 to *P* < 1E-6. See Additional file 3 for mean single-site methylation data in tabular form.

In SRS patients S1 to S3 and S5, ICR1 was significantly hypomethylated in all three tissues examined when compared with the controls K1 to K3. The ICR1 hypomethylation was strongest in blood leukocytes (25 versus 56%; *P* < 0.001), moderately less severe in oral mucosa cells (32 versus 60%; *P* < 0.001), and mildest in cultured skin fibroblasts (43 versus 54%; *P* = 0.022) (Table 2 and Figure 2A). In contrast, ICR2 methylation was normal in all tissues of the SRS patients (see Additional file 2).

**Table 2:** **Imprinting center region** 1 **(ICR1) methylation levels in patients with Silver-Russell syndrome (SRS) and controls**

	**Blood**	**Buccal smears**		**Skin fibroblasts**	
**SRS patients**		**Short side**^**a**^	**Long side**	**Short side**	**Long side**
S1	18^b^ ± 2 (4)^c^	32 ± 4 (3)	33 ± 4 (4)	40 ± 2 (3)	48 ± 5 (3)
S2	25 ± 2 (3)	25 ± 1 (3)	25 ± 3 (3)	38 ± 1 (2)	30 ± 1 (2)
S3	36 ± 2 (3)	43 ± 1 (3)	46 ± 2 (3)	48 ± 5 (5)	58 ± 1 (2)
S4	21 ± 2 (4)	28 ± 1 (2)	35 ± 5 (2)	nd^d^	nd
S5	27 ± 3 (5)	34 ± 3 (2)	23 ± 1 (2)	36 ± 1 (2)	44 ± 1 (2)
Mean S1 to S5	25 ± 5^e^	32 ± 7	32 ± 9	41 ± 5	45 ± 12
		32^f^ ± 8		43^g^ ±9	
**Controls**		L	R	L	R
K1	52 ± 2 (2)	64 ± 0 (2)	69 ± 2 (2)	51 ± 2 (2)	nd
K2	53 ± 5 (2)	53 ± 3 (3)	55 ± 2 (4)	56 ± 3 (2)	57 ± 4 (2)
K3	64 ± 4 (3)	61 ± 5 (2)	60 ± 4 (2)	53 ± 1 (2)	52 ± 2 (2)
Mean K1 to K3	56 ± 5	60 ± 6		54 ± 3	

^a^For location of short side see Table 1; ^b^MS-MLPA determined methylation level in% (100% = fully methylated); ^c^number of MS-MLPA determinations in brackets; ^d^nd = not done; ^e^*P* (S1 to S5: K1 to K3)_blood_ = 0.00077; ^f^*P* (S1 to S5: K1 to K3)_buccal smears_ = 0.0000024; ^g^*P* (S1 to S5: K1 to K3)_skin fibroblasts_ = 0.022. L, left side; R, right side.

When comparing the methylation values measured in tissues from the two asymmetric halves of the body, there was no clear-cut difference observed. In oral mucosa cells, mean ICR1 methylation values from the short and the long side of the asymmetric body of the SRS patients were comparable (Table 2). In fibroblasts from the shorter forearm, ICR1 hypomethylation was more severe in three out of four SRS patients (S1, S3, and S5), but not in patient S2 (Table 2).

### Hypomethylation of single CpG sites within ICR1

The above used mean from the CpG sites M1 to M5 does not reflect the whole complexity of the ICR1 hypomethylation observed in SRS. Therefore, we calculated CpG site-specific methylation values as well (Figure 2B).

Only sites M2 and M3 were significantly hypomethylated in all tissues from SRS patients investigated. Sites M1 and M4 were found to be significantly hypomethylated in blood leukocytes and oral mucosa cells, but not in cultured fibroblasts. Site M5 was normally methylated in all tissues examined.

More detailed analysis showed that in blood leukocytes and oral mucosa cells of all five SRS patients M1 to M3 methylation levels were well below the control range (see Additional file 4), while in fibroblasts some values not only for M1, but also for M2 and M3 methylation, were in the range of the controls. There was no correlation between the degree of hypomethylation in bilateral oral mucosa or skin fibroblasts probes and body asymmetry, at any sites in the relevant SRS patients (data not shown).

### ICR1 methylation pattern of clones originating from a single fibroblast

The above shown variable degrees of hypomethylation are thought to reflect a mosaicism of cells carrying absent, low, or normal levels of methylation of the CpG sites M1 to M4. Therefore, isolation and propagation of one single cell out of this cell mosaic should extinguish mosaicism and enable the characterization of the methylation pattern within this single cell.

The patterns of single CpG site hypomethylation in clonal cultures from patient S2 showed substantial variability. In some of the cell clones from patient S2, the degree of hypomethylation at the CpG sites M2 to M4 was almost 0%, while other clones from the same patient showed normal methylation values at these sites (Table 3). CpG site M1 was also demethylated in most of the control clones tested, corresponding to the low M1 methylation level of the culture of origin, control patient (K1), left side (L). The methylation level of site M5 was not different between clones from SRS patient S2 and from the control patient.

**Table 3:** M1 to M5 methylation levels of clonal fibroblast cultures from Silver-Russell syndrome (SRS) patient S2 and control patient K1

**Fibroblast culture**	**Origin/clone**	**n**^**a**^	**% Methylation of individual MS-MLPA sites**				
			**M1**	**M2**	**M3**	**M4**	**M5**
S2L (short side)	origin	2	4^b^ ± 3	13 ± 8	17 ± 6	62 ± 10	97 ± 6
	1	2	26 ± 8	2 ± 1	45 ± 5	47 ± 1	114 ± 7
	2	2	66 ± 10	1 ± 1	58 ± 7	66 ± 2	118 ± 5
	3	2	1 ± 1	0 ± 0	6 ± 1	43 ± 5	90 ± 4
	4	2	34 ± 3	0 ± 0	49 ± 7	44 ± 8	100 ± 3
S2R (long side)	origin	2	1 ± 1	3 ± 0	15 ± 6	31 ± 5	100 ± 1
	1	2	0 ± 0	0 ± 0	5 ± 2	0 ± 0	99 ± 13
	2	2	1 ± 1	0 ± 0	10 ± 2	44 ± 1	107 ± 4
	3	2	0 ± 0	0 ± 0	1 ± 1	2 ± 2	84 ± 13
	4	2	0 ± 0	4 ± 4	11 ± 1	6 ± 1	78 ± 7
K1L	origin	2	17 ± 7	35 ± 8	43 ± 11	70 ± 6	92 ± 21
	1	3	0 ± 0	44 ± 3	59 ± 8	44 ± 7	120 ± 10
	2	3	0 ± 0	51 ± 6	51 ± 5	75 ± 10	99 ± 8
	3	2	1 ± 1	33 ± 10	44 ± 5	49 ± 1	99 ± 10
	4	2	0 ± 0	44 ± 2	43 ± 5	60 ± 7	82 ± 5
	5	3	38 ± 2	45 ± 6	49 ± 1	43 ± 9	100 ± 19
	6	3	1 ± 0	28 ± 6	61 ± 7	81 ± 7	94 ± 9
	7	2	1 ± 1	26 ± 1	39 ± 2	102 ± 4	91 ± 3

^a^Number of MS-MLPA determinations; ^b^MS-MLPA determined methylation level in%. MS-MLPA, methylation-specific multiple ligation-dependent probe amplification; L, left; R, right.

### *IGF2* expression in cultured skin fibroblasts from SRS patients

**Figure 3:**
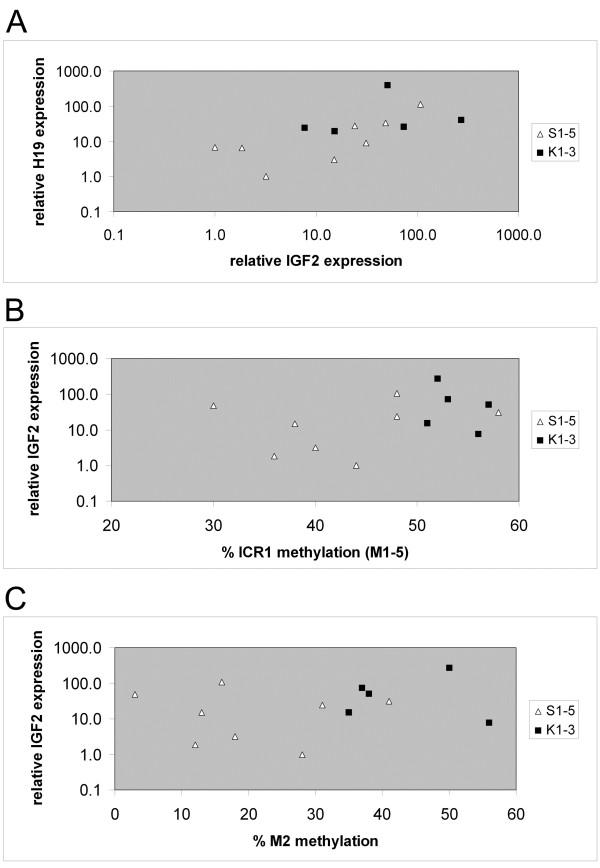
***IGF2 *and *H19 *expression in skin fibroblast cultures.***IGF2* and *H19* mRNA levels were determined by real time (RT)-PCR. See Additional file 6 for data in tabular form. (**A**) *IGF2* and *H19* expression values from the individual cultures are related. Virtually no correlation was present for the control cultures (r_Pearson_ = −0.12) and a positive correlation for the Silver-Russell syndrome (SRS) cultures (r_Pearson_ = 0.96). (**B**) The *IGF2* expression values are related to the imprinting center region 1 (ICR1) methylation value (mean of MS-MLPA sites M1 to M5) from the same culture (Table 2). (**C**) The *IGF2* expression values are related to the methylation values from site M2 only (see Additional file 4 for tabular methylation data).

Relative *IGF2* and *H19* mRNA levels in fibroblasts varied over a broad range. Control cultures showed poor quantitative correlation of *IGF2* and *H19* mRNA levels (Figure 3A), while in SRS cultures there was a positive correlation of both mRNA analytes. An *H19* mRNA excess or an *IGF2* mRNA deficiency in SRS patients compared to controls was not evident. The degree of ICR1 hypomethylation (mean of M1 to M5 or M2 alone) did not predict the amount of *IGF2* mRNA produced (Figures 3B and 3C). Control experiments (priming cDNA synthesis with random hexamers instead of oligo dT) resulted in comparable results (see Additional file 5) with some larger variations in the microRNA precursor *H19* mRNA. Also, experiments using a different PCR primer pair for the amplification of *IGF2* cDNA conformed to the reported measurements (see Additional file 5).

### IGF-II secretion of cultured skin fibroblasts

Levels of secreted IGF-II were not significantly different (*P* > 0.6) between fibroblast cultures from SRS patients (mean 5.4 ± 2.3 ng/ml) and controls (5.0 ± 1.0 ng/ml) (Table 4). Fibroblasts obtained from the shorter arm were associated with lower IGF-II concentrations in two patients, and in the other 2 patients, with higher IGF-II concentrations than fibroblasts obtained from the longer arm of the respective individual.

**Table 4:** IGF-II concentrations in supernatant from cultured skin fibroblasts

	**IGF-II (ng/ml)**	
**SRS patients**	**Short side**^**a**^	**Long side**
S1	5.6^b^ ± 0.4	4.7 ± 0.2
S2	8.2 ± 1.1	6.4 ± 0.1
S3	3.4 ± 0.3	8.8 ± 0.9
S5	2.6 ± 0.1	3.4 ± 0.3
Mean S1 to S5	4.9^c^ ± 2.5	5.8 ± 2.3
	5.4 ±2.3	
**Controls**	**L**	**R**
K1	6.4 ± 0.2	nd^d^
K2	5.1 ± 1.8	3.9 ± 0.6
K3	5.4 ± 0.8	4.1 ± 0.4
Mean K1 to K3	5.0 ±1.0	

^a^For location of short body side see Table 1; ^b^mean of two experiments; ^c^*P*-value > 0.6 for comparison between short side and long side, as well as for comparison between SRS and control groups; ^d^nd = not done. SRS, Silver-Russell syndrome; L, left; R, right.

### Basal and stimulated cell proliferation of cultured skin fibroblasts

**Figure 4:**
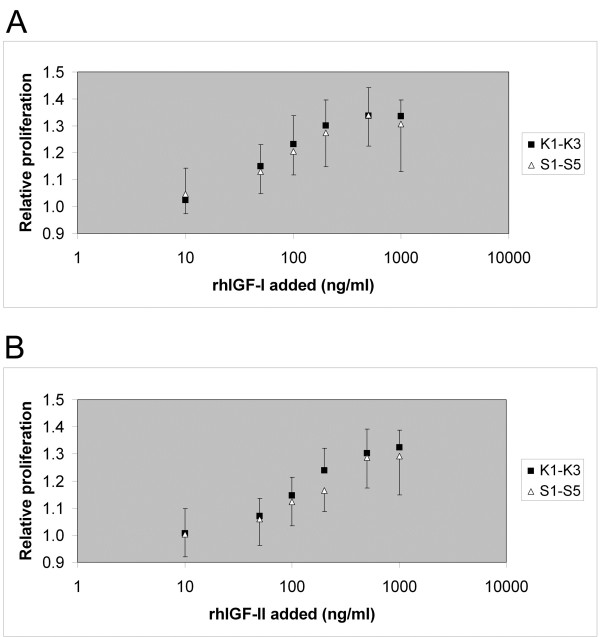
**IGF-I and IGF-II stimulated proliferation of skin fibroblasts.** Skin fibroblasts grown 4 days in culture were treated with various concentrations of recombinant IGF-I (**A**) and IGF-II (**B**) until (3-(4,5-dimethylthiazol-2-yl)-5-(3-carboxymethoxyphenyl)-2-(4-sulfophenyl)-2 H-tetrazolium, inner salt (MTS) test at day 7. Relative proliferation values were calculated by normalization to cultures treated with diluent (culture medium) only. For IGF-I stimulation, mean EC50 values of 81 ± 30 ng/ml (S1 to S5) and 62 ± 25 ng/ml (K1 to K3) were obtained; *P*_IGF-I_ (S_1-5_:K_1-3_) = 0.246. For IGF-II stimulation, mean EC50 values of 168 ± 96 ng/ml (S1 to S5) and 114 ± 33 ng/ml (K1 to K3) were obtained; *P*_IGF-II_ (S_1-5_:K_1-3_) = 0.261. See Additional files 7 and 8 for tabular data. S, patients with Silver-Russell syndrome; K, control patients.

Basal fibroblast proliferation rates in standard culture medium were tested at low and high initial cell densities and for different periods of time after seeding the cells. There were no significant differences between SRS patient fibroblasts and control fibroblasts (Table 5). Stimulating fibroblast proliferation by adding recombinant human IGF-I or IGF-II, respectively, to the culture medium resulted in comparable dose response curves (Figure 4) with no significant differences regarding EC50 values or proliferation responses at the diverse hormone concentrations tested.

**Table 5:** Basal proliferation of skin fibroblast cultures

**Number of seeded cells**	**OD**_**d4**_**/OD**_**d1**_^**a**^			**OD**_**d7**_**/OD**_**d4**_^**b**^		
	**S1 to S5**	**K1 to K3**	***T*- test**	**S1 to S5**	**K1 to K3**	***T*- test**
	**Mean (n = 16)**^**c**^	**Mean (n = 10)**^**d**^	***P***	**Mean (n = 16)**^**c**^	**Mean (n = 10)**^**d**^	***P***
200 cells/well	4.25 ± 2.04	5.33 ± 1.67	0.17	2.41 ± 1.33	2.09 ± 0.74	0.49
1600 cells/well	2.92 ± 1.22	3.54 ± 1.01	0.19	1.36 ± 0.26	1.33 ± 0.40	0.81

^a^Mean absorption from (3-(4,5-dimethylthiazol-2-yl)-5-(3-carboxymethoxyphenyl)-2-(4-sulfophenyl)-2 H-tetrazolium, inner salt (MTS) test after 4 days in culture divided by that after 1 day in culture; ^b^mean absorption from MTS test after 7 days in culture divided by that after 4 days in culture; ^c^two tests done for each of the cultures from patients S1, S2, S3, and S5 L/R; ^d^two tests done for each of the cultures from K1L, K2, and K3 L/R. S, patient with Silver-Russell syndrome; K, control patient; L, left side; R, right side.

## Discussion

Studying five asymmetrically growing adolescent children with SRS in comparison to three control probands we observed a strong tissue-dependence of the degree of ICR1 hypomethylation within the *IGF2/H19* locus. Body asymmetry was not correlated with a different degree of ICR1 hypomethylation found in tissue probes taken from both sides of the body. Detailed methylation analysis revealed differential hypomethylation of the five examined ICR1 CpG sites in all patients. Analysis of cultured skin fibroblasts after clonal expansion confirmed the complex cellular mosaicism of the ICR1 hypomethylation. *IGF2* expression, IGF-II secretion, and cell proliferation of cultured fibroblasts from SRS patients were not different to controls and without a significant difference in samples taken from the two asymmetric body halves of the same patient.

ICR1 hypomethylation was most severe in blood leukocytes, less severe in oral mucosa cells, and least severe in cultured skin fibroblasts. It is unlikely that the moderate ICR1 hypomethylation in fibroblasts was due to effects of cell culture on ICR1 methylation, that is, a preferential loss of fibroblasts with ICR1 hypomethylation during culture or some general healing effect of cell culture on ICR1 hypomethylation. First, clonal fibroblast cultures prepared from original cultures showed virtually absent methylation for several ICR1 CpG sites. Second, another study [8] reported an even stronger hypomethylation in cultured skin fibroblasts of one SRS patient compared to blood leukocytes. Third, two recent microarray studies [32,33] gave no evidence that the methylation of two ICR1 CpG sites is significantly different between young (passages 2 to 3) and long-term (passages 21 to 48) skin fibroblast cultures overall from five normal subjects (see Additional file 9 for data and links to the relevant datasets), indicating that normal ICR1 methylation is not affected by skin fibroblast cell culture.

Reports on the various degrees of hypomethylation in different tissues in SRS are inconsistent. As already mentioned above, Gicquel *et al*. [8] found a more severe hypomethylation in cultured skin fibroblasts than in blood leukocytes in a single patient with SRS. Yamazawa *et al*. [28] reported on a stronger hypomethylation in blood leukocytes than in preserved placental tissues from three patients. Begemann *et al*. [13] observed a more severe hypomethylation in blood leukocytes than in buccal smears in two SRS patients and the opposite for one additional patient. These contrasting findings may reflect different experimental settings or the presence of real individual characteristics of the mosaic hypomethylation. In the majority of the reported cases, however, the degree of ICR1 hypomethylation was positively correlated to the degree of the proliferation rate of the specific tissue. If this holds true for a larger group of patients, it may indicate that the mechanism of hypomethylation (defective maintenance of normal methylation or defective repair of aberrant methylation) can be overcome by slow proliferation.

The methylation of the ICR2 within the *IGF2*/*H19* locus was found to be unchanged in all tissues from SRS patients, confirming results from previous studies in which concomitant ICR1 and ICR2 hypomethylation was shown to be a rare event [12,13].

Three-quarters of the SRS patients with ICR1 hypomethylation present with body asymmetry (76% [9]; 71% [7]; 77% [11]; 68% [3]). Our hypothesis of a mosaicism with a higher degree of ICR1 hypomethylation at the shorter body side could not be confirmed in bilaterally-obtained probes of oral mucosa and skin. Our observation renders it unlikely that the postnatal persistence of asymmetric growth in the adolescent SRS patients [4] is due to a persisting long side-short side ICR1 hypomethylation difference. Epigenetic mechanisms causing body asymmetry may be restricted to the intrauterine development.

The ICR1 CpG sites M1 to M4 were differentially hypomethylated in the three tissues examined from SRS patients. This and the different patterns of demethylated and normally methylated sites in clonal skin fibroblast cell lines indicate the presence of a complex cellular mosaic in SRS, consisting of multiple different cells in terms of the number and position of CpG sites affected from hypomethylation within the ICR1. Interestingly, tissue-dependent variability of methylation was found also in control patients for some CpG sites (M1, M5). The last finding emphasizes the importance of methods with specificity for single CpG sites to clearly define the extent of hypomethylation in SRS patients.

Information on *IGF2* and *H19* expression in tissue from SRS patients is scarce. In cultured skin fibroblasts from two SRS patients [8] and in paraffin-embedded placental tissue from three SRS fetuses [28] a correlation of ICR1 hypomethylation with suppressed *IGF2* expression was shown. In contrast, we observed no ICR1 hypomethylation-dependent reduction of *IGF2* expression in cultured skin fibroblasts from SRS patients. Possible explanations for this discrepancy are outlined as follows.

The major difference of the study by Gicquel *et al*. [8] to ours is the different degree of the ICR1 hypomethylation of the SRS patients investigated. The cultured skin fibroblasts of our four SRS patients exhibited a mean ICR1 methylation level of 43%, ranging from 30 to 58% according to MS-MLPA. In contrast, Gicquel *et al*. [8] studied cultured skin fibroblasts from a single SRS patient with an ICR1 methylation level (determined by southern blot analysis) of only 10% and from a second patient with that same very low methylation level in blood leukocytes (corresponding value for fibroblasts was not reported). Therefore, ICR1 hypomethylation in cultured skin fibroblasts of SRS patients may only be functionally relevant in terms of reducing *IGF2* expression when falling below a certain threshold not reached in the individual specimens collected for our study. This interpretation is supported by the finding that the clinical phenotype in SRS patients with ICR1 hypomethylation is more severe in the presence of a very low methylation level (< 9%) than in patients with a less pronounced ICR1 hypomethylation [11].

In the other study linking ICR1 hypomethylation to decreased *IGF2* expression, RNA was extracted from paraffin-embedded placental tissues from three pregnancies with SRS fetuses [28]. In contrast to our study, the ICR1 methylation indices were determined with the combined bisulfite restriction analysis (COBRA). In addition, one has to note that RNA quantification from paraffin-embedded placental tissues is technically difficult, potentially influencing the results [28]. The measured placental ICR1 methylation indices were at 30 to 33% and therefore at the lower end of the ICR1 methylation levels for cultured skin fibroblasts from SRS patients in our study. The relevant methylation-sensitive restriction site was juxtaposed to site M2 from our study. The reduced *IGF2* expression for the hypomethylated placental specimens found by Yamazawa *et al*. [28] may indicate a tighter ICR1 control of *IGF2* expression in the placenta than in the cultured skin fibroblasts we investigated. In line with this, appropriate *IGF2* expression and therefore also its regulation is well known to be of pivotal importance for normal placental development [18,34].

A model in which the ICR1 methylation status reciprocally regulates *IGF2* and *H19* expression [22-24,35] was not supported by our data, since we did not find a corresponding inverse correlation of the two mRNA analytes. In accordance with this, others have experimentally shown that the binding of the insulator protein CTCF to unmethylated ICR1, suggested to be mechanistically linked to downregulation of *IGF2* expression and upregulation of *H19* expression, did not result in the predicted gene expression in diverse human tissues [36]. Also, in one of the studies already mentioned above [28], *IGF2* suppression in placental specimens from SRS patients was not accompanied by enhanced *H19* expression. Clearly, more tissue-related experiments will be necessary to further understand cellular downstream effects of the ICR1 hypomethylation in SRS patients. Theoretically, changes in so far unknown regulatory elements acting at the *IGF2*/*H19* locus in addition to ICR1 methylation, including other chromatin modifications, may exist in SRS patients. A further level of regulation would potentially also explain the large variations we found in *IGF2* and *H19* expression in controls.

Normal to high IGF-I and IGF-II serum levels in SRS patients with ICR1 hypomethylation [6,7] in comparison to age- and stature-related references hinted at some degree of IGF-I insensitivity. In a single case report [37] on cultured skin fibroblasts from a SRS patient with ICR1 hypomethylation and extraordinary high IGF-I serum level, a reduced proliferative response to IGF-I stimulation compared to controls was found. In contrast, we found normal proliferation of fibroblasts from four SRS patients in response to IGF-I and IGF-II stimulation. These results render more unlikely the hypothesis that decreased IGF-I sensitivity is a leading cause of growth failure in SRS.

Potential limitations of our study are as follows. The SRS group in this study consisted of five patients (three female, two male) and was compared with three control subjects (all male), and some caution regarding possible gender differences, albeit not observed by us, should remain. In addition, the number of individuals examined in our study was limited by the fact that biopsy material, including skin specimens, is difficult to obtain from juvenile individuals. Finally, for the study of the growth deficit in SRS the availability of bone tissue would be desirable, but bone biopsy however, would only be possible in the very rare SRS patients undergoing bone surgery for medical reasons.

## Conclusions

ICR1 hypomethylation in SRS patients is tissue- and CpG site-dependent and shows a complex cellular mosaicism. Asymmetric growth in SRS children is not related to a more pronounced ICR1 hypomethylation at the shorter side of the body, rendering more likely, intrauterine developmental defects as causative for asymmetry. An influence of ICR1 hypomethylation on *IGF2* expression in SRS may only become evident in the case of a strong hypomethylation and may be more prevalent in placental tissue than in the cultured skin fibroblasts from the adolescent patients we investigated. An unaltered fibroblast proliferation response to exogenous IGF-I and IGF-II do not support the hypothesis of IGF-I insensitivity in SRS. The establishment of clonal cell cultures is likely to overcome some of the inherent obstacles in studying the effects of an epigenetic mutation expressed as a cellular mosaic.

### Availability of supporting data

The data sets supporting the results of this article are included within the article (and its additional files).

## Methods

### Individuals

The protocol of the study was reviewed by the Ethics Committee of the Medical Faculty of Tübingen. All patients, controls, and parents of patients and controls gave their written informed consent before enrolment.

The five SRS patients with ICR1 hypomethylation (identified as S1 to S5; 9.2 to 16.6 years of age, two male) were recruited at our Pediatric Endocrinology Outpatient Department. Body asymmetry was documented by photography of the hands of each patient. Clinical characteristics of the patients are shown in Table 1.

Three healthy children (identified as K1 to K3; all male; 14.0, 14.7, and 15.0 years of age) served as controls. These children were recruited at our Pediatric Surgery Department where they underwent operation under anesthetic for removal of metals or wires after arm fracture healing.

A blood sample and two buccal smears from both cheeks were obtained from all individuals. Skin punch biopsies (2 mm punches from Stiefel, Offenbach, Germany) from both forearms were obtained from patients S1, S2, S3 and S5, and from the controls K2 and K3. From control K1, a skin biopsy was obtained only from one arm. No skin biopsy was obtained from patient S4.

### Genomic DNA preparation

Genomic DNA was prepared from whole blood (Genomic DNA Extraction Kit; Macherey & Nagel, Düren, Germany), buccal smears (Puregene Buccal Cell Core kit; Qiagen, Hilden, Germany), and cultured fibroblasts (DNeasy Tissue Kit; Qiagen).

### Methylation analysis by MS-MLPA

The methylation status of the ICR1 of the *IGF2*/*H19* locus and of the ICR2 in the *KCNQ1* gene was measured with the Salsa MLPA kit ME030-B2 (MRC-Holland, Amsterdam, Netherlands) [38-40]. The MS-MLPA method [38] employed for the kit is a modification of the original MLPA technique [39,40]. Specifically, MS-MLPA kit ME030 was designed to detect and quantify abnormal methylation ratios of the two imprinted domains H19DMR (ICR1) and KvDMR (ICR2) as well as deletions and duplications of one or more exons of the 11p15 genes. The basis of the method is the quantitative PCR amplification of target DNA after restriction with a DNA methylation-sensitive enzyme (HhaI) specific for the CpG-containing sequence GCGC. HhaI cleaves only unmethylated DNA, while a GC_methyl_GC sequence is resistant to cleavage. For each investigated CpG site, a PCR product of specific length surrounding the site is obtained.

In each MS-MLPA reaction, five different sites named M1 to M5 (Figure 1 and Additional file 1) from the ICR1 and four different sites from the ICR2 (Additional file 1) were analyzed simultaneously. For each reaction, 200 ng of genomic DNA was used. The extent of methylation at a specific site (expressed in%) could be calculated from reference PCR reactions for genomic sequences lacking a GCGC tetranucleotide and from a parallel reaction of the same, but unrestricted genomic DNA sample. The mean of the methylation values from M1 to M5 was taken to characterize ICR1 methylation as a whole, and correspondingly ICR2 methylation was calculated as the mean of the methylation values from the four *KCNQ1* ICR2 sites. MS-MLPA results for ICR1 methylation correspond well with a bisulfite conversion-based method for DNA methylation analysis using the Humanmethylation27 bead array [31].

### Fibroblast cell culture

Fibroblast cell cultures were established in RPMI (Gibco) + 10% FCS (Biochrom, Berlin, Germany) + 100 units/ml penicillin/streptomycin (Biochrom). In brief, 2 mm skin punch biopsies were manually dissected into five to ten tissue pieces and transferred to 1.5 ml medium in a 25-cm^2^ cell culture flask (BD Falcon, Heidelberg, Germany). After 8 to 10 days of culture, outgrowth of fibroblasts from tissue pieces adherent to the flask bottom was microscopically observed. At that time 5.5 ml medium was added, and cultivation was continued. After approximately 5 to 6 weeks of culture, cells reached confluence and were trypsinized and passaged to 75-cm^2^ flasks. Further passages were done with 1:4 cell dilutions. Fibroblasts from passages 4 to 8 were used for experiments aimed at the quantification of ICR1 methylation levels, secreted factors, specific mRNAs, cell proliferation, or the generation of clonal cell lines. All experiments were done in culture medium as above. Fibroblast cultures were named according to the body side of origin (S2L for example indicates the culture obtained from the biopsy taken at the left arm of patient S2).

Starting from a single cell out of the cultured skin fibroblast, clonal cultures for SRS patient S2 and for control individual K1 were generated. Single cells from strongly diluted solutions of trypsinized fibroblasts were transferred with a micropipette under microscopic control into single wells of 24 or 48 well plates (BD Falcon). Online microscopic monitoring ensured that only one cell was aspirated during the process of single cell picking. Cell clones reaching confluence were trypsinized and cultivated in larger cell containers. Cell aliquots from clone passage 4, corresponding to roughly 18 to 20 cell divisions (2x10^5^ to 10^6^ cells) after single cell picking, were used for DNA methylation analysis. No outgrowth of cells was observed when cultivating medium volumes devoid of cells aspirated from the diluted cell suspensions.

### *IGF2* and *H19* mRNA quantification

*IGF2* and *H19* mRNA were quantified by real-time PCR from reverse-transcribed whole fibroblast RNA. Trypsinized fibroblasts were seeded at a density of 5,400 cells/cm^2^ in 75-cm^2^ cell culture flasks (BD Falcon). After 7 days in culture, whole cellular RNA was prepared from cells directly lysed in lysis buffer (Qiagen RNeasy kit) including 10% β-mercaptoethanol. After reverse transcription (250 ng whole RNA per reaction, oligo dT priming; Invitrogen Superscript III kit) *IGF2* and *H19* transcripts were quantified by real-time PCR (IQ Sybr Green Supermix from Bio-Rad, München, Germany; Bio-Rad CFX0096 cycler) with glyceraldehyde 3-phosphate dehydrogenase (GAPDH) normalization. Primer design was done with NCBI Primer Blast. See Additional file 10 for sequences of primers used.

### IGF-II measurements in culture medium

Secretion of IGF-II by fibroblasts into the culture medium was monitored with an in-house radioimmunoassay (RIA) (range 0.31 to 40 ng/ml, intra/inter assay variation 8/8.6%). In each experiment, the culture medium not incubated with the fibroblasts was also measured to determine and subtract the background level due to crossreacting bovine IGF-II which was found to be at 3.5 to 5.3 ng/ml.

Trypsinized fibroblasts were seeded at a cell density of 6,250/cm^2^, corresponding to 60,000 cells/3 ml medium/well of a 6-well plate (Falcon). After 1 day in culture, the medium was changed. After 7 days in culture, supernatant probes were taken and assessed as described above. Mean values of 2 to 3 wells/culture were determined.

### Fibroblast proliferation

To measure baseline proliferation rates under normal culture conditions, fibroblasts were seeded at 200 cells/well (low cell density, 713 cells/cm^2^) or 1,600 cells/well (high cell density, 5,700 cells/cm^2^) of three 96-well plates and incubated for 1, 4, or 7 days. Cell proliferation was determined with the MTS test [41](Promega, Madison, WI, USA) for one plate after 1 day in culture and for the parallel plates after 4 and 7 days in culture, respectively, by photometry (absorption measurement at 490 nm with reference wavelength 630 nm). Cell-free medium wells incubated in parallel served as negative controls for background values. Absorption ratios day_4_/day_1_ and day_7_/day_4_ were calculated to determine proliferation rates. Mean values of 3 wells/point/culture were calculated. For each fibroblast culture, two experiments were performed.

To measure the extent of the stimulation of fibroblast proliferation by IGF-I and IGF-II, different concentrations of rhIGF-I and rhIGF-II (Gropep, Adelaide, Australia) diluted in culture medium were added on day 4 to wells seeded on day 0 as described above. MTS test and photometry followed on day 7. Values for the relative proliferative effect of the different hormone concentrations were determined by dividing the values for the rhIGF-I/rhIGF-II-stimulated fibroblasts through those for fibroblasts treated with diluent only and grown in parallel. Mean EC50 values for SRS and control cultures were determined with help of the Sigma Plot software.

### Statistical analysis

For the levels of DNA methylation, *IGF2* and *H19* mRNA expression, and IGF-II protein amount, mean deviations were calculated from multiple determinations (relevant numbers given in the corresponding tables). For mean values of subject groups, SD was calculated. Statistical comparison between subject groups was done by two-sided *t*-tests. *P*-values < 0.05 were regarded to be significant.

## Abbreviations

BLAST: basic local alignment search tool; COBRA: combined bisulfite restriction analysis; CTCF: CCCTC-binding factor; FCS: fetal calf serum; GAPDH: glyceraldehyde-3-phosphate dehydrogenase; ICR1: imprinting center region 1; IGF1: insulin-like growth factor 1; IGFBP-3: insulin-like growth factor binding protein 3; KCNQ1: potassium voltage-gated channel KQT-like subfamily, member 1; MS-MLPA: methylation-specific multiple ligation-dependent probe amplification; MTS: (3-(4,5-dimethylthiazol-2-yl)-5-(3-carboxymethoxyphenyl)-2-(4-sulfophenyl)-2 H-tetrazolium inner salt; PCR: polymerase chain reaction; rhIGF-II: recombinant human insulin-like growth factor 2; RIA: radioimmunoassay; SRS: Silver-Russell syndrome.

## Competing interests

The authors declare that they have no competing interests.

## Authors’ contributions

KK and GB conceived the project and drafted the manuscript. KK designed the experiments. GB took the skin punch biopsies. KW carried out most of the fibroblast culture experiments. CB carried out the real-time PCR experiments. CU carried out the MS-MLPA determinations. Skin punch biopsies of control individuals were obtained under the supervision of HJK. All authors read and approved the final manuscript.

## Supplementary Material

Additional file 1 Description: A table showing the genomic positions of CpG sites investigated by methylation-specific multiple ligation-dependent probe amplification (MS-MLPA) and of CTCF binding sites 1–7. Click here for file

Additional file 2 Description: A table showing imprinting center region (ICR)2 methylation levels in Silver-Russell syndrome (SRS) patients and controls. Click here for file

Additional file 3 Description: A table showing mean methylation levels of individual imprinting center region 1 (ICR1) sites M1 to M5 in Silver-Russell syndrome (SRS) patients and controls. Click here for file

Additional file 4 Description: A table showing methylation levels of individual imprinting center region 1 (ICR1) sites M1 to M5 in Silver-Russell syndrome (SRS) patients and controls. Click here for file

Additional file 5 Description: A figure showing control measurements for IGF2 and H19 expression. Click here for file

Additional file 6 Description: A table showing relative IGF2 and H19 mRNA levels in skin fibroblast cultures from Silver-Russell syndrome (SRS) patients and controls. Click here for file

Additional file 7 Description: A table showing IGF-I stimulated proliferation of skin fibroblasts. Click here for file

Additional file 8 Description: A table showing IGF-II stimulated proliferation of skin fibroblasts. Click here for file

Additional file 9 **Description: A table showing values for methylation of imprinting center region 1 (ICR1) CpG sites in early and late passages of skin fibroblast cultures obtained from Koch*et al*. [32,33].** Click here for file

Additional file 10 **Description:A table showing the primers used for RT-PCR quantification of*IGF2*and*H19*mRNAs.** Click here for file
