# Supplementary material for: IGF2/H19 hypomethylation is tissue, cell, and CpG site dependent and not correlated with body asymmetry in adolescents with Silver-Russell syndrome
Source: Clin Epigenetics. 2012 Sep 18;4(1):15. doi: 10.1186/1868-7083-4-15 (PMC3523983; doi:10.1186/1868-7083-4-15)
Supplement: Additional file 1 — Description: A table showing the genomic positions of CpG sites investigated by methylation-specific multiple ligation-dependent probe amplification (MS-MLPA) and of CTCF binding sites 1–7. [file 1868-7083-4-15-S1.pdf]

Additional File 1: Genomic positions of CpG sites investigated by MS-MLPA and of CTCF binding sites 1-7

MS-MLPA probe ID	Gene symbol	Position in GRCH37:11	Name	Position in gene[a]
6266-L5772	*H19*	2019397[b]	M1	upstream *H19* exon 1
11080-L11762	*H19*	2019566	M2	upstream *H19* exon 1
8744-L8764	*H19*	2019737	M3	upstream *H19* exon 1
8743-L8763	*H19*	2020030	M4	upstream *H19* exon 1
		2020275-2020288[c]		CTCF binding site 7
8745-L8765	*H19*	2020496	M5	upstream *H19* exon 1
		2021198-2021111		CTCF binding site 6
		2021604-2021617		CTCF binding site 5
		2022010-2022023		CTCF binding site 4
		2024244-2024257		CTCF binding site 3
		2024244-2024257		CTCF binding site 2
		2024249-2024262		CTCF binding site 1
7171-L6780	*KCNQ1*	2720587	/	*KCNQ1* intron 10
6276-L5782	*KCNQ1*	2720649	/	*KCNQ1* intron 10
7172-L6781	*KCNQ1*	2721056	/	*KCNQ1* intron 10
7173-L6782	*KCNQ1*	2721437	/	*KCNQ1* intron 10

a: Exon sequences obtained from NR_002196.1 (*H19*) and NM_000218.2 (*KCNQ1*) and matched to the genomic sequence by BLAST search

b: Genomic location of MS-MLPA sites according to BLAST search with the source sequence provided by MRC Holland against human genome build GRCh37 from the Genome Reference Consortium accessible at http://www.ensembl.org

c: Genomic location of CTCF binding sites according to BLAST search with the CTCF site sequences provided in Bell et al.[22] against human genome build GRCh37 from the Genome Reference Consortium accessible at http://www.ensembl.org
