# Supplementary material for: IGF2/H19 hypomethylation is tissue, cell, and CpG site dependent and not correlated with body asymmetry in adolescents with Silver-Russell syndrome
Source: Clin Epigenetics. 2012 Sep 18;4(1):15. doi: 10.1186/1868-7083-4-15 (PMC3523983; doi:10.1186/1868-7083-4-15)
Supplement: Additional file 2 — Description: A table showing imprinting center region (ICR)2 methylation levels in Silver-Russell syndrome (SRS) patients and controls. [file 1868-7083-4-15-S2.pdf]

Additional File 3: ICR2 methylation levels in SRS patients and controls

	Blood	Buccal smears		Skin fibroblasts	
**SRS**		short side	long side	short side	long side
S1	55[a] ±4 (4)[b]	58 ±6 (3)	57 ±5 (4)	53 ±3 (3)	63 ±7 (3)
S2	53 ±1 (3)	57 ±2 (3)	53 ±2 (3)	49 ±5 (2)	62 ±0 (2)
S3	59 ±6 (3)	50 ±2 (3)	55 ±4 (3)	55 ±5 (5)	51 ±2 (2)
S4	51 ±2 (4)	59 ±0 (2)	62 ±0 (2)	nd[c]	nd
S5	53 ±3 (5)	56 ±5 (2)	55 ±8 (2)	48 ±3 (2)	49 ±2 (2)
Mean S1-S5	54 ±3	56 ±4	56 ±3	51 ±3	56 ±7
		56 ±3		54 ±6	
**controls**		L	R	L	R
K1	51 ±4 (2)	56 ±3 (2)	59 ±1 (2)	41 ±4 (2)	nd
K2	51 ±2 (2)	44 ±3 (3)	50 ±6 (4)	50 ±2 (2)	49 ±0 (2)
K3	58 ±3 (3)	52 ±5 (2)	53 ±5 (2)	53 ±4 (2)	50 ±1 (2)
Mean K1-K3	53 ±4	52 ±5		49 ±5	

a: MS-MLPA determined methylation level in % (100% = fully methylated)

b: Number of MS-MLPA determinations in brackets

c: Nd = not done
