# Supplementary material for: IGF2/H19 hypomethylation is tissue, cell, and CpG site dependent and not correlated with body asymmetry in adolescents with Silver-Russell syndrome
Source: Clin Epigenetics. 2012 Sep 18;4(1):15. doi: 10.1186/1868-7083-4-15 (PMC3523983; doi:10.1186/1868-7083-4-15)
Supplement: Additional file 3 — Description: A table showing mean methylation levels of individual imprinting center region 1 (ICR1) sites M1 to M5 in Silver-Russell syndrome (SRS) patients and controls. [file 1868-7083-4-15-S3.pdf]

Additional File 4: Mean methylation levels of individual ICR1 sites M1-5 in SRS patients and controls

		M1[a]	M2	M3	M4	M5
Blood	S1-S5 (*n*=5)	12[b] ±7	13 ±6	17 ±4	17 ±10	68 ±9
	K1-K3 (*n*=3)	49 ±9	52 ±3	56 ±8	47 ±9	78 ±8
	P	<0.001	<1E-4	<0.001	<0.01	ns[c]
Buccal smears	S1-S5 (*n*=10)[d]	14 ±9	16 ±7	23 ±7	37 ±16	71 ±12
	K1-K3 (*n*=6)[d]	46 ±3	46 ±7	61 ±10	65 ±17	82 ±9
	P	<1E-6	<1E-6	<1E-6	<0.01	ns
Skin fibroblasts	S1-S5 (*n*=8)[e,f]	14 ±12	20 ±12	29 ±12	54 ±14	97 ±6
	K1-K3 (*n*=5)[e,g]	26 ±7	43 ±9	48 ±7	54 ±14	98 ±6
	P	ns	<0.01	<0.01	ns	ns

a: For location of CpG sites M1-5 see Fig. 1 and Additional File 1

b: Mean MS-MLPA determined methylation level in % (100% = site fully methylated)

c: Ns = not significant

d: Probes from both cheeks of each individual

e: Fibroblast cultures from both arms

f: No skin fibroblasts available from patient S4

g: From patient K1 only one fibroblast culture available
