# Supplementary material for: IGF2/H19 hypomethylation is tissue, cell, and CpG site dependent and not correlated with body asymmetry in adolescents with Silver-Russell syndrome
Source: Clin Epigenetics. 2012 Sep 18;4(1):15. doi: 10.1186/1868-7083-4-15 (PMC3523983; doi:10.1186/1868-7083-4-15)
Supplement: Additional file 4 — Description: A table showing methylation levels of individual imprinting center region 1 (ICR1) sites M1 to M5 in Silver-Russell syndrome (SRS) patients and controls. [file 1868-7083-4-15-S4.pdf]

Additional File 5: Methylation levels of individual ICR1 sites M1-5 in SRS patients and controls

			n[a]	M1[b]	M2	M3	M4	M5
Blood	S1		4	4[c] ±3	5 ±3	12 ±3	5 ±1	62 ±4
	S2		3	16 ±2	16 ±2	16 ±2	18 ±2	62 ±4
	S3		3	18 ±1	19 ±1	24 ±2	33 ±3	84 ±8
	S4		4	6 ±3	10 ±5	16 ±2	11 ±8	66 ±1
	S5		5	16 ±2	16 ±2	19 ±3	19 ±4	65 ±8
	K1		2	43 ±3	54 ±9	54 ±10	37 ±8	71 ±6
	K2		2	44 ±1	48 ±6	49 ±5	49 ±11	77 ±13
	K3		3	60 ±6	53 ±7	65 ±13	55 ±8	87 ±12
Buccal smears	S1	L	4	12 ±8	18 ±7	21 ±8	40 ±7	74 ±9
		R	3	7 ±6	10 ±5	30 ±7	21 ±10	94 ±14
	S2	L	3	14 ±4	13 ±1	17 ±3	29 ±4	49 ±3
		R	3	12 ±5	15 ±1	18 ±2	25 ±4	57 ±4
	S3	L	3	23 ±2	19 ±2	29 ±3	65 ±3	78 ±4
		R	3	30 ±3	31 ±3	35 ±2	59 ±4	76 ±4
	S4	L	2	11 ±8	12 ±0	23 ±1	51 ±20	78 ±15
		R	2	3 ±1	12 ±2	22 ±1	35 ±4	69 ±9
	S5	L	2	24 ±1	23 ±1	29 ±6	22 ±11	72 ±1
		R	2	3 ±1	8 ±2	12 ±9	21 ±15	70 ±2
	K1	L	2	44 ±16	57 ±1	57 ±3	85 ±16	79 ±1
		R	2	47 ±14	46 ±2	72 ±2	85 ±6	96 ±1
	K2	L	3	49 ±4	46 ±4	45 ±2	53 ±8	72 ±10
		R	4	47 ±9	48 ±9	59 ±7	46 ±8	74 ±6
	K3	L	2	49 ±0	41 ±0	60 ±8	55 ±9	84 ±15
		R	2	42 ±7	37 ±2	72 ±5	65 ±1	87 ±18
Fibroblasts	S1	L	3	23 ±6	16 ±11	42 ±11	74 ±10	86 ±20
		R	3	7 ±4	18 ±2	28 ±1	44 ±3	101 ±6
	S2	L	2	4 ±3	13 ±8	17 ±6	62 ±10	97 ±6
		R	2	1 ±1	3 ±0	15 ±6	31 ±5	100 ±1
	S3	L	5	27 ±7	31 ±4	39 ±16	52 ±10	92 ±9
		R	2	31 ±2	41 ±3	45 ±3	70 ±12	105 ±2
	S5	L	2	2 ±1	12 ±7	17 ±1	48 ±5	102 ±17
		R	2	18 ±2	28 ±1	29 ±3	51 ±5	96 ±7
	K1	L	2	17 ±7	35 ±8	43 ±11	70 ±6	92 ±21
	K2	L	2	29 ±5	56 ±2	56 ±5	35 ±2	105 ±17
		R	2	33 ±3	38 ±10	53 ±9	66 ±5	95 ±7
	K3	L	2	22 ±18	37 ±5	49 ±2	52 ±0	103 ±27
		R	2	32 ±1	50 ±7	39 ±8	47 ±1	95 ±9

a: Number of MS-MLPA determinations

b: For location of CpG sites M1-5 see Fig. 1 and Additional File 1

c: MS-MLPA determined methylation level in % (100% = site fully methylated)
