# Supplementary material for: IGF2/H19 hypomethylation is tissue, cell, and CpG site dependent and not correlated with body asymmetry in adolescents with Silver-Russell syndrome
Source: Clin Epigenetics. 2012 Sep 18;4(1):15. doi: 10.1186/1868-7083-4-15 (PMC3523983; doi:10.1186/1868-7083-4-15)
Supplement: Additional file 5 — Description: A figure showing control measurements for IGF2 and H19 expression. [file 1868-7083-4-15-S5.pdf]

Additional File 7: Control measurements for IGF2 and H19 expression

A

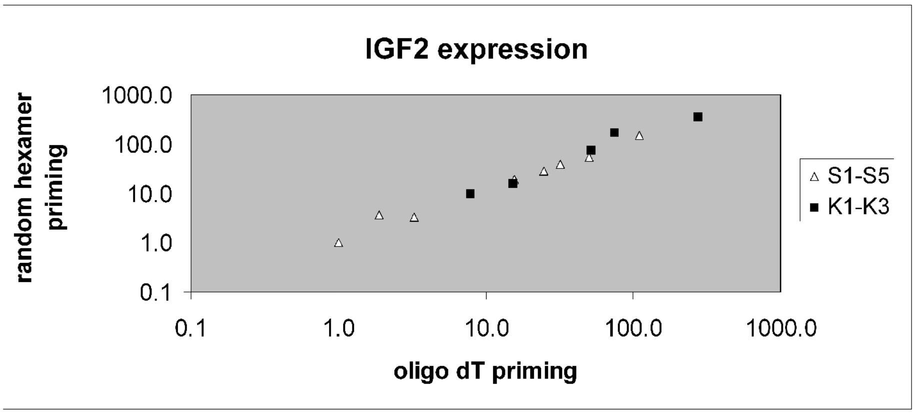

B

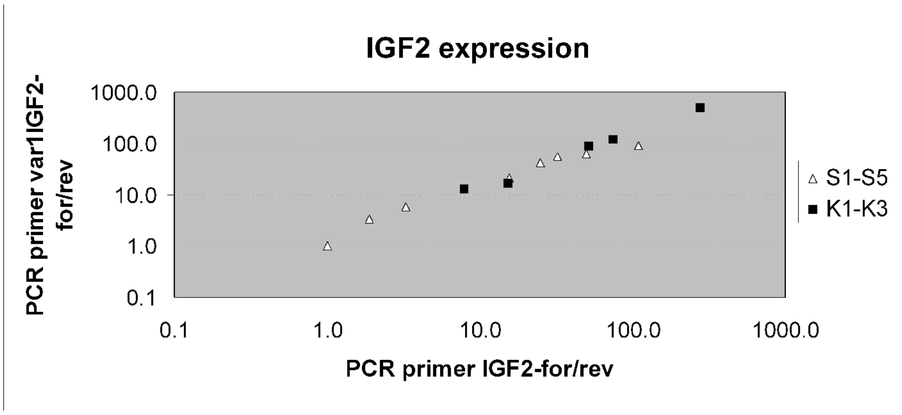

C

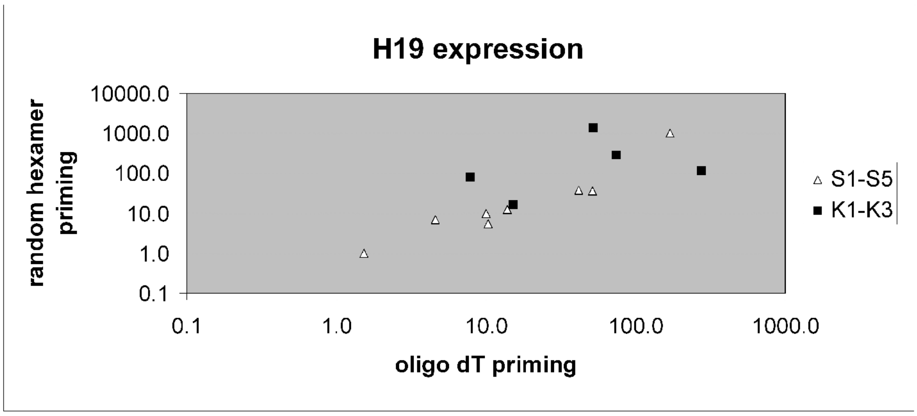

Legend Additional File 7. Control measurements for *IGF2* and *H19* expression.

A. X-axis: *IGF2* expression measured with oligo dT primed cDNA (corresponding to Fig. 2 and Additional File); y-axis: *IGF2* expression measured with random hexamer primed

cDNA. **B.** X-axis: *IGF2* expression measured with PCR primer pair IGF2-for/rev (corresponding to Fig. 2 and Additional File); y-axis: *IGF2* expression measured with PCR primer pair var1IGF2-for/rev (for primer sequence see Additional File 2). **C.** X-axis: *H19* expression measured with oligo dT primed cDNA (corresponding to Fig. 2A and Additional File 6); y-axis: *H19* expression measured with random hexamer primed cDNA.
