# Supplementary material for: IGF2/H19 hypomethylation is tissue, cell, and CpG site dependent and not correlated with body asymmetry in adolescents with Silver-Russell syndrome
Source: Clin Epigenetics. 2012 Sep 18;4(1):15. doi: 10.1186/1868-7083-4-15 (PMC3523983; doi:10.1186/1868-7083-4-15)
Supplement: Additional file 6 — Description: A table showing relative IGF2 and H19 mRNA levels in skin fibroblast cultures from Silver-Russell syndrome (SRS) patients and controls. [file 1868-7083-4-15-S6.pdf]

Additional File 6: Relative *IGF2* and *H19* mRNA levels in skin fibroblast cultures from SRS patients and controls

	***IGF2* mRNA**		***H19* mRNA**		**Ratio *IGF2/H19***	
**SRS patients**	short side[a]	long side	short side	long side	short side	long side
S1	3.27[b] ±0.00	109.74 ±0.47	1.52 ±0.00	169.86 ±5.48	2.15	0.65
S2	15.55 ±0.15	49.97 ±1.22	4.59 ±0.00	51.14 ±1.01	3.39	0.98
S3	24.75 ±0.04	32.11 ±0.31	41.69 ±0.09	13.87 ±0.03	0.59	2.32
S5	1.88 ±0.00	1.00 ±0.00	9.95 ±0.03	10.28 ±0.02	0.19	0.10
**Mean S1-S5**	11.36[c] ±10.83	48.20 ±45.74	14.44[c] ±18.50	61.28 ±74.70	1.58[c] ±1.47	1.01 ±0.94
	29.78[c] ±36.53		37.86[c] ±56.26		1.29[c] ±1.18	
**controls**	L	R	L	R	L	R
K1	15.33 ±0.14	nd[d]	29.57 ±0.09	nd	0.52	nd
K2	7.87 ±0.02	51.93 ±36.47	36.47 ±0.55	596.25 ±77.38	0.22	0.09
K3	74.67 ±0.01	277.05 ±18.63	38.98 ±0.11	62.59 ±1.77	1.92	4.43
**Mean K1-K3**	85.37 ±110.55		152.77 ±248.22		1.43 ±1.82	

a: For location of short body side see Table 1

b: GAPDH-normalized relative *IGF2* and *H19* mRNA expression, mean of two experiments; for easier comparison the overall lowest value (*IGF2* expression of fibroblasts from S5 long side) was set to 1 assuming comparable target amplification efficiencies in the PCR reactions

c: All *P* values > 0.2 for comparisons short side:long side as for comparisons SRS patients:controls

d: Nd = not done
