# Supplementary material for: IGF2/H19 hypomethylation is tissue, cell, and CpG site dependent and not correlated with body asymmetry in adolescents with Silver-Russell syndrome
Source: Clin Epigenetics. 2012 Sep 18;4(1):15. doi: 10.1186/1868-7083-4-15 (PMC3523983; doi:10.1186/1868-7083-4-15)
Supplement: Additional file 7 — Description: A table showing IGF-I stimulated proliferation of skin fibroblasts. [file 1868-7083-4-15-S7.pdf]

Additional File 8: IGF-I stimulated proliferation of skin fibroblasts

Cultures	rhIGF-I added (ng/ml) after 4 days in culture					
	**10**	**50**	**100**	**200**	**500**	**1000**
S1-S5 (L/R; n=8)	1.05[a] ±0.07	1.13 ±0.08	1.21 ±0.09	1.27 ±0.13	1.34 ±0.11	1.31 ±0.18
K1-K3 (L/R; n=5)	1.02 ±0.12	1.15 ±0.08	1.23 ±0.10	1.30 ±0.09	1.34 ±0.10	1.34 ±0.06
P	0.69	0.68	0.62	0.68	0.99	0.73

a: MTS test measured absorption after 7 days in culture normalized to mock-treated cultures grown in parallel
