# Supplementary material for: IGF2/H19 hypomethylation is tissue, cell, and CpG site dependent and not correlated with body asymmetry in adolescents with Silver-Russell syndrome
Source: Clin Epigenetics. 2012 Sep 18;4(1):15. doi: 10.1186/1868-7083-4-15 (PMC3523983; doi:10.1186/1868-7083-4-15)
Supplement: Additional file 8 — Description: A table showing IGF-II stimulated proliferation of skin fibroblasts. [file 1868-7083-4-15-S8.pdf]

Additional File 9: IGF-II stimulated proliferation of skin fibroblasts

Cultures	rhIGF-II added (ng/ml) after 4 days in culture					
	**10**	**50**	**100**	**200**	**500**	**1000**
S1-S5 (L/R; n=8)	1.00[a] ±0.08	1.06 ±0.10	1.12 ±0.09	1.16 ±0.08	1.29 ±0.11	1.29 ±0.14
K1-K3 (L/R; n=5)	1.01 ±0.09	1.07 ±0.07	1.15 ±0.07	1.24 ±0.08	1.30 ±0.09	1.32 ±0.06
P	0.95	0.85	0.61	0.12	0.78	0.65

a: MTS test measured absorption after 7 days in culture normalized to mock-treated cultures grown in parallel
