# Supplementary material for: IGF2/H19 hypomethylation is tissue, cell, and CpG site dependent and not correlated with body asymmetry in adolescents with Silver-Russell syndrome
Source: Clin Epigenetics. 2012 Sep 18;4(1):15. doi: 10.1186/1868-7083-4-15 (PMC3523983; doi:10.1186/1868-7083-4-15)
Supplement: Additional file 9 — Description: A table showing values for methylation of imprinting center region 1 (ICR1) CpG sites in early and late passages of skin fibroblast cultures obtained from Kochet al. [32,33]. [file 1868-7083-4-15-S9.pdf]

Additional File 10: Values for methylation of ICR1 CpG sites in early and late passages of skin fibroblast cultures obtained from Koch et al. [30, 31]

Subject	Methylation (beta values)[a] of ICR1 sites			
	Array site cg17769238[b]		Array site cg02657360[c]	
	Early passage	Late passage	Early passage	Late passage
Donor15[d]	Passage 3	Passage 21	Passage 3	Passage 21
	0.550	0.606	0.765	0.781
III[e]	Passage 2	Passage 39	Passage 2	Passage 39
	0.649	0.605	0.740	0.618
VI[f]	Passage 2	Passage 33	Passage 2	Passage 33
	0.622	0.617	0.644	0.694
VII[g]	Passage 2	Passage 34	Passage 2	Passage 34
	0.641	0.591	0.660	0.624
VIII[h]	Passage 2	Passage 31	Passage 2	Passage 31
	0.640	0.587	0.639	0.672
Mean	0.620 ±0.040	0.601 ±0.012	0.689 ±0.058	0.678 ±0.066
P (early:late)	0.411		0.732	

a: Beta values obtained from measurements with Infinium Humanmethylation27 BeadChip (Illumina); beta: continuous variable from 0-1 corresponding to 0-100% methylation

b: Investigated CpG site located at Chr11:2019568, 2 bp distant from MS-MLPA site M2[39]

c: Investigated CpG site located at Chr11:2020560, 64 bp distant from MS-MLPA site M5 [39]

d: Beta values obtained from datasets GSM560606 (HDF-otoplastic_donor15_6yrs_P3) and GSM560607 (HDF-otoplastic_donor15_6yrs_P21); GEO accession number GSE22595 [30]

e: Beta values obtained from datasets GSM735504 (Fib_D_III_abdomen_P2_62) and GSM735505 (Fib_D_III_abdomen_P39_62); GEO accession number GSE29661 [31]

f: Beta values obtained from datasets GSM735506 (Fib_D_VI_breast_P2_43) and GSM735507 (Fib_D_VI_breast_P33_43); GEO accession number GSE29661 [31]

g: Beta values obtained from datasets GSM735508 (Fib_D_VII_leg_P2_48) and GSM735509 (Fib_D_VII_leg_P34_48); GEO accession number GSE29661 [31]

h: Beta values obtained from datasets GSM735510 (Fib_D_VIII_abdomen_P2_43) and GSM735511 (Fib_D_VIII_abdomen_P31_43); GEO accession number GSE29661 [31]
