# Supplementary material for: IGF2/H19 hypomethylation is tissue, cell, and CpG site dependent and not correlated with body asymmetry in adolescents with Silver-Russell syndrome
Source: Clin Epigenetics. 2012 Sep 18;4(1):15. doi: 10.1186/1868-7083-4-15 (PMC3523983; doi:10.1186/1868-7083-4-15)
Supplement: Additional file 10 — Description:A table showing the primers used for RT-PCR quantification ofIGF2andH19mRNAs. [file 1868-7083-4-15-S10.pdf]

Additional File 2: Primers for RT-PCR quantification of *IGF2* and *H19* mRNAs

Primer	Sequence	Length
IGF2-for	CAGTGAGACCCTGTGCGGCG	20
IGF2-rev	TCCCTCTCGGACTTGGCGGG	20
H19-for	TGAGCTCTCAGGAGGGAGGATGGT	24
H19-rev	TTGTCACGTCCACCGGACCTG	21
GAPDH-for	TGCACCACCAACTGCTTAGC	20
GAPDH-rev	GGCATGGACTGTGGTCATGAG	21
var1IGF2-for	CTGCCCCGTCGCACATTCGG	20
var1IGF2-rev	ATTGGTGTCTGGAAGCCGGCGA	22
